# Evaluation of non-gastric upper gastrointestinal system polyps: an epidemiological assessment

**DOI:** 10.1038/s41598-023-33451-1

**Published:** 2023-04-15

**Authors:** Çağdaş Erdoğan, Derya Arı, Bayram Yeşil, Kenan Koşar, Orhan Coşkun, İlyas Tenlik, Hasan Tankut Köseoğlu, Mahmut Yüksel

**Affiliations:** Department of Gastroenterology, Ankara City Hospital, University of Health Sciences, Bilkent Avenue, Çankaya, 06800 Ankara, Turkey

**Keywords:** Gastroenterology, Gastrointestinal system, Oesophagogastroscopy

## Abstract

Non-gastric upper gastrointestinal system polyps are detected rarely and mostly incidentally during upper gastrointestinal endoscopy. While the majority of lesions are asymptomatic and benign, some lesions have the potential to become malignant, and may be associated with other malignancies. Between May 2010 and June 2022, a total of 127,493 patients who underwent upper gastrointestinal endoscopy were retrospectively screened. Among these patients, those who had polyps in the esophagus and duodenum and biopsied were included in the study. A total of 248 patients with non-gastric polyps were included in this study. The esophageal polyp detection rate was 80.00/100,000, while the duodenal polyp detection rate was 114.52/100,000. In 102 patients (41.1%) with esophageal polyps, the mean age was 50.6 ± 15.1, and 44.1% (n = 45) were male. The most common type of polyps was squamous papilloma (n = 61, 59.8%), followed by inflammatory papilloma (n = 18, 17.6%). In 146 patients (58.9%) with duodenal polyps, the mean age of patients was 58.3 ± 16.5, and 69.8% (n = 102) were male. Brunner's gland hyperplasia, inflammatory polyp, ectopic gastric mucosa, and adenomatous polyp were reported to be the most prevalent types of polyps in the duodenum overall (28.1%, 27.4%, 14.4%, and 13.7%, respectively). It is crucial to identify rare non-gastric polyps and create an effective follow-up and treatment plan in the era of frequently performed upper gastrointestinal endoscopies. The epidemiological assessment of non-gastric polyps, as well as a follow-up and treatment strategy, are presented in this study.

## Introduction

Upper gastrointestinal endoscopy (esophagogastroduodenoscopy, EGD) includes evaluation of the oropharynx, esophagus, stomach, and proximal duodenum. EGD can be performed with indications such as dyspeptic complaints unresponsive to medical treatment, presence of alarm symptoms, upper gastrointestinal symptoms after the age of 50, dysphagia, persistent vomiting, or upper gastrointestinal bleeding. Polyps are mostly detected incidentally during upper gastrointestinal endoscopy. However, management and appropriate pathological evaluation of polyps are very important^1,2^.

**Figure 1:**
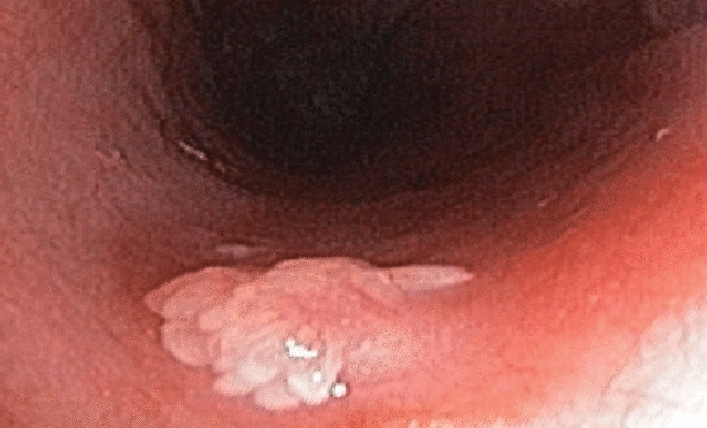
Esophageal squamous papilloma.

Many benign lesions can be encountered during the endoscopic evaluation of the esophagus. Most lesions are rare and asymptomatic. Although most of these lesions do not have malignant potential, some lesions can be considered as premalignant. Since glycogenic acanthosis, the most prevalent polypoid lesion in the esophagus, has a frequency of 3.5–15%, a characteristic structure, and a benign nature, these lesions are simple to identify and don't need to be biopsied or evaluated pathologically^3–5^. With a rate between 0.01% and 0.45%, esophageal squamous papilloma (Fig. 1) are relatively the most prevalent polypoid lesions in the esophagus^6,7^. It is mostly seen in patients around 50 years of age, in the distal esophagus and as a single lesion^8^. Although most papilloma are asymptomatic, dysphagia due to large papilloma has been reported rarely^9^. Esophageal papillomas are followed in incidence by inflammatory polyps^10^, esophageal parakeratosis^11^, and esophageal adenomas^12–14^ that develop on the basis of Barrett’s esophagus and carry malignant potential.

Lymphangiomas^15^ and neuroendocrine tumors that originate from the submucosa are other esophageal lesions that can come across. These lesions can, however, only be found extremely rare. Neuroendocrine tumors can be seen in the pancreas or tubular organs of the GI system and show neuroendocrine differentiation. Endoscopically, neuroendocrine tumors of the digestive tract can present as polypoid forms, nodules, masses, ulcers, or stenosis, and they can be single or multiple and range in size from a few millimeters to several centimeters. These tumors, which are rare in the esophagus (only 50 cases have been documented), typically form sessile polypoid structures in the lower third^16^.

**Figure 2:**
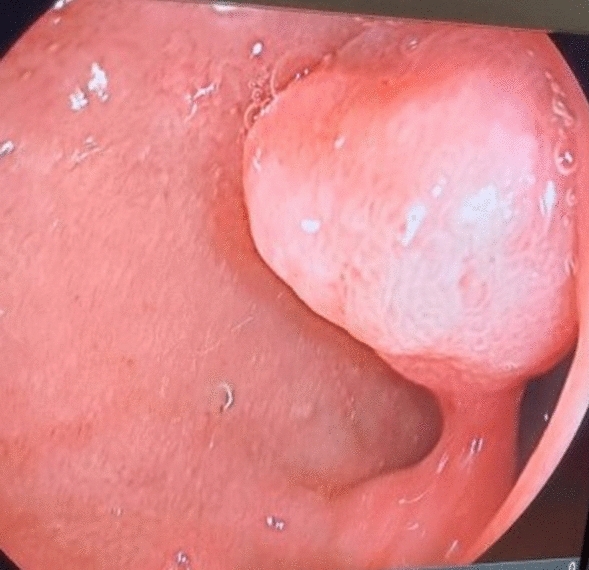
Duodenal adenomatous polyp.

**Figure 3:**
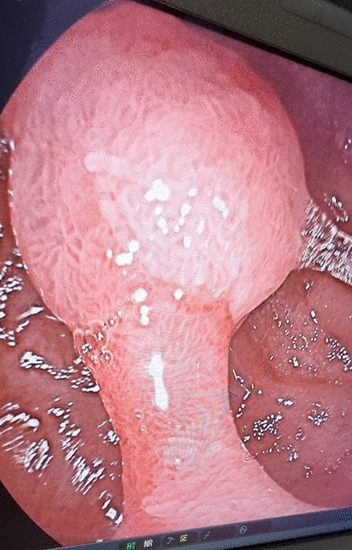
Duodenal adenomatous polyp magnified.

Duodenal polyps are generally quite rare and can be classified as non-neoplastic and neoplastic. Based on the respective incidence, non-neoplastic lesions include ectopic gastric mucosa, inflammatory polyps, Brunner's gland hyperplasia, peutz-jeghers polyps, and hyperplastic polyps. Whereas, neoplastic lesions include adenomas, gastrointestinal stromal tumors, Brunner's gland adenoma, carcinoid tumors, leiomyoma, lipoma, schwannoma can be counted. Duodenal adenomas (Figs. 2, 3) have three major types: villous adenomas, tubular adenomas, and Brunner's gland adenomas. Villous adenomas carry a significant risk of malignancy. Since the incidence of colon adenomas increases in patients with duodenal polyps, colonoscopy should be performed when these polyps are detected^17^.

Tubular adenomas are more common in the duodenum, are mostly asymptomatic and have less malignant potential. Brunner's gland adenomas are rare small intestinal polyps that are more common, accounting for 10.6% of duodenal tumors^18^. Ectopic gastric mucosa may present as polypoid lesions which are rare congenital disorders and are detected incidentally during upper GI endoscopy. It has been reported in the literature that heterotopic gastric mucosa may be associated with duodenal ulcers^19^. Gastrointestinal stromal tumors (GIST) are mostly encountered in the stomach, they can also be seen in the esophagus (< 1%) and duodenum (5%)^20^. Tumors originating from the upper GI may present with dysphagia, GI bleeding, or obstructive jaundice.

Duodenal or ampullary NETs are extremely rare and account for approximately 2.6% of all NETs^21^. These tumors are of clinical importance as most of them are asymptomatic and potentially malignant. They typically occur in the I and II duodenal sections, preferring the peripapillary region, and under endoscopic vision, they show as a single, small lesion (frequently less than 1 cm in size). Additionally, they may exist in groups or be linked to neuroendocrine tumors in other organs^16^.

In this study we aimed to evaluate the epidemiological distribution of polyps detected during EGD and submitted to pathological assessment by biopsy, as well as the follow-up and treatment strategy in polyps with malignant potential or symptomatic.

## Patients/material and methods

Our study was approved by Ankara City Hospital Scientific Research Evaluation and Ethics Committee (Approval No: E1-22-2328). The procedures implemented until February 2019 were carried out at Ankara Turkey Yüksek İhtisas Training and Research Hospital. Since Ankara Turkey Yüksek İhtisas Training and Research Hospital joined the Ankara City Hospital after February 2019, the patients included in the study after this date were selected among the patients followed up and treated at Ankara City Hospital.

Between May 2010 and June 2022, a total of 127,493 patients who underwent upper gastrointestinal endoscopy with indications such as dyspepsia, dysphagia, and iron deficiency anemia were retrospectively screened. Among these patients, those who had polyps in the esophagus and duodenum and biopsied were included in the study.

In patients who underwent EGD, biopsy or excision was performed on all polyps detected in the esophagus that were solitary or larger than 1 cm. All patients with Barrett's esophagus discovered to have polyps underwent biopsies. When dysplasia clusters are observed rather than a single polyp formation in Barrett’s esophagus, which is where the majority of esophageal adenomas arise from, the vascular pattern was assessed with NBI endoscopy, and a biopsy was collected for esophageal adenoma. In addition, biopsies or excisions were performed on hyperemic polyps, polyps with aberrant vascular patterns on narrow-band imaging (NBI) endoscopy, and ulcerated polyps. However, no biopsy was performed when multiple instances of glycogenic acanthosis were found. In case of detection of polyps in the duodenum, polyps were biopsied or excised from all patients.

Patients who had polyps but could not be biopsied due to antiaggregant/anticoagulant use, hemodynamic instability, and patient intolerance were excluded from the study. In addition, patients with gastric polyps were also excluded. Additionally, patients with esophageal polyps who underwent biopsies and who, upon pathologic inspection, revealed to have glycogenic acanthosis were removed from the study.

Patients’ demographic characteristics such as age, gender, smoking, history of comorbid diseases and drug use were recorded. Polyp sizes, polyp types, number of polyps and histopathological findings were recorded in patients who were found to have esophageal and duodenal polyps and biopsied. Additionally, antrum biopsies were performed to check for *H. pylori* in patients with polyps. The pathologists stained the tissue samples with Giemsa and tested for the presence of *H. pylori*. Patients with helicobacter pylori positivity as a result of pathology were separately identified. According to the pathology results, whether the patients had reflux findings in their histories, whether they had a history of head and neck cancer, and previous endoscopic and colonoscopy findings, if any, were evaluated. The frequency per 100,000 of esophageal and duodenal polyps detected in the patients was reported. The pathological distributions of the polyps were also displayed as a rate per 100,000.

In patients undergoing colonoscopy the cleanliness of the colonoscopy was evaluated using the Boston Bowel Preparation Scale (BBPS). Following colonoscopy cleaning, patients with BBPS 0 or 1 were taken for another colonoscopy. Colonoscopy cleanliness scores BBPS 2 and 3 were used to assess all study participants who had non-gastric polyps. Withdrawal from the colonoscopy took at least 10 min.

Endoscopic evaluation was performed with Olympus brand GIF-Q260 model gastroscopes. Before the procedure, patients were given sedo-analgesia or topical anesthetic containing 10% lidocaine to the oropharynx, accompanied by an anesthesiologist. The lesions were removed with forceps or snare. The biopsy material was fixed with 10% formaldehyde solution and sent for pathological evaluation.

### Statistical analysis

All statistical analyzes were performed using SPSS software (SPSS for Windows, version 25.0, IBM. Corp., Armonk, NY, USA). The Kolmogorov–Smirnov test was used to determine the normality of the continuous variables. Normally distributed variables were expressed as mean ± standard deviation and non-normally distributed variables as median and interquartile range. Normally distributed variables were compared using the student t test and non-normally distributed variables using the Mann–Whitney U test. Chi-square (χ^2^) test and Fisher's Exact test were used for group comparisons (cross tables) of nominal variables. Two-tailed p values < 0.05 were considered statistically significant.

### Ethics committee approval

This study was complied with the ethical guidelines of the 1975 Helsinki Declaration that was then modified in 2008. The study protocol was approved by Ankara City Hospital ethics committee (Approval No: E1-22-2328).

### Informed consent

Informed consent was obtained from all patients participating in the study.

## Results

A total of 248 patients who were evaluated for non-gastric polyps were included in this study. Total evaluation revealed a detection rate of 194.52 non-gastric polyps per 100,000 patients. In this context, the esophageal polyp detection rate was 80.00 per 100,000, while the duodenal polyp detection rate was 114.52 per 100,000.

One hundred and two (41.1%) of the included patients were those with esophageal polyps. The mean age of patients with esophageal polyps was 50.6 ± 15.1, and 44.1% (n = 45) were male. The median size of esophageal polyps was 5.0 (4.0–7.0) mm. Pedunculated polyps were seen in 63.7% of cases (n = 65), while sessile polyps were found in 36.3% of cases (n = 37). The most common type of polyps was squamous papilloma (n = 65, 63.7%), followed by inflammatory papilloma (n = 18, 17.6%). Helicobacter pylori was found in 26 (25.5%) and reflux esophagitis in 21 (20.7%) patients. The most common endoscopic gastric finding was pangastritis (n = 49, %48.0) followed by antral gastritis (n = 31, %30.4). Adenomatous polyps were detected in 15 of 76 patients with esophageal polyps who underwent colonoscopy. Table 1 includes demographic information as well as endoscopic and colonoscopic findings, pathology results, and epidemiological rates of patients with esophageal polyps.

**Table 1:** Demographic characteristics, endoscopic and colonoscopy findings, and pathological evaluations of patients with esophageal polyps, pathologic findings as rate per 100,000 patients.

	All patients (n = 102)	Rate per 100,000 patients
Age, years	50.6 ± 15.1	–
Gender, male, n (%)	45 (44.1)	–
Smoking, n (%)	56 (54.9)	–
Polyp size, mm	5.0 (4.0–7.0)	–
Number of polyps	1.0 (1.0–1.0)	–
Polyp type, n (%)		
Pedunculated	65 (63.7)	–
Sessile	37 (36.3)	–
Pathology, n (%)		
Squamous papilloma	65 (63.7)	50.98
Inflammatory polyp	21 (20.7)	16.47
Hyperplastic polyp	3 (2.9)	2.35
Lymphangioma	3 (2.9)	2.35
Esophageal parakeratosis	6 (5.9)	4.70
Esophageal adenoma	4 (3.9)	3.14
*Helicobacter pylori*, n (%)	26 (25.5)	–
Reflux esophagitis, n (%)	17 (16.7)	–
Other endoscopic findings, n (%)		
Normal	8 (7.8)	–
Antral gastritis	31 (30.4)	–
Bulbitis	3 (2.9)	–
Pangastritis	49 (48.0)	–
Antral gastritis + bulbitis	9 (8.9)	–
Gastric lymphoma	2 (2.0)	–
Colonoscopy, n (%)	76 (74.5)	–
Colon polyp localization, n (%)		
Transverse + ascending + sigmoid	3 (2.9)	–
Transverse + ascending + rectum	3 (2.9)	–
Descending + transverse	4 (3.9)	–
Rectum	5 (4.9)	–
Colon polyp pathology, n (%)		
Adenomatous	15 (14.7)	–

Data are expressed as median ± SD or median (IQR) or frequency (%).

*SD* standard deviation.

When patients with duodenal polyps were evaluated (n = 146), polyps were observed in the duodenal bulb in 91 patients (62.3%) and in the second part of the duodenum (D2) in 55 patients (37.7%). The mean age of patients with duodenal polyps was 58.3 ± 16.5, and 69.8% (n = 102) were male. The median size of duodenal polyps was 7.0 (3.5–15.0) mm. Brunner's gland hyperplasia, inflammatory polyp, ectopic gastric mucosa, and adenomatous polyp were reported to be the most prevalent types of polyps in the duodenum overall (28.1%, 27.4%, 14.4%, and 13.7%, respectively). In subgroup analysis Brunner's gland hyperplasia was most common in bulbus (36%), while adenomatous polyp was most common in D2 (bulbus vs. D2; 2 vs. 18; p < 0.001). All polyps with adenomatous pathology were detected in the second continent of the duodenum, whereas individuals with ectopic gastric mucosa as a result of pathology had polyp localization in the first continent (p0.00.1 for both findings). The presence of antral gastritis, bulbitis, duodenitis, pangastritis, pangastritis + bulbitis, atrophic gastritis, FAP, gastric cancer and peutz-jeghers syndrome was at similar rates between both groups. In addition, colonoscopy was performed in 88 (%60.3) patients with polyps in the duodenum, and polyps were detected in the colon in 35 (%24) of them. While hyperplastic polyp was found in the colon in two patients with polyp in the bulbus, adenomatous polyp was detected in the colon in the 33 patients and in all patients with polyps in the second part of the duodenum. Table 2 includes demographic information as well as endoscopic and colonoscopic findings, pathology results, and epidemiological rates of patients with duodenal polyps.

**Table 2:** Demographic characteristics, endoscopic and colonoscopy findings, and pathological evaluations of patients with duodenal polyps, pathologic findings as rate per 100,000 patients.

	All patients (n = 146)	Rate per 100,000 patients
Age, years	58.3 ± 16.5	–
Gender, male, n (%)	102 (69.8)	–
Smoking, n (%)	81 (55.5)	–
Polyp size, mm	7.0 (3.5–15.0)	–
Number of polyps	1.0 (1.0–2.5)	–
Polyp localization, n (%)		
Duodenal bulb	91 (62.3)	71.38
Second part of the duodenum (D2)	55 (37.7)	43.14
Pathology, n (%)		
Brunner gland hyperplasia	41 (28.1)	32.16
Ectopic gastric mucosa	21 (14.4)	16.47
Inflammatory polyp	40 (27.4)	31.37
Villous adenoma	4 (2.7)	3.14
Neuroendocrine tumor	3 (2.1)	2.35
Tubular adenoma	4 (2.7)	3.14
Hyperplastic polyp	7 (4.8)	5.49
Adenomatous polyp	20 (13.7)	15.69
Hamartomatous polyp	6 (4.1)	4.71
*Helicobacter pylori*, n (%)	14 (9.6)	–
Other endoscopic findings, n (%)		
Antral gastritis	50 (34.2)	–
Duodenal ulcer	13 (8.8)	–
Duodenitis	3 (2.1)	–
Pangastritis	64 (43.8)	–
Pangastritis + bulbitis	5 (3.4)	–
Atrophic gastritis	3 (2.1)	–
FAP	3 (2.1)	–
Gastric CA	3 (2.1)	–
Peutz-jeger	2 (1.4)	–
Colonoscopy, n (%)	88 (60.3)	
Detection of polyps in colonoscopy, n (%)	35 (24.0)	–
Colon polyp localization, n (%)		
Rectosigmoid	22 (15.1)	–
Other colonic parts	13 (8.9)	–
Polyp type, n (%)		
Hyperplastic	2 (1.4)	–
Adenomatous	33 (22.6)	–

Data are expressed as median ± SD or median (IQR) or frequency (%).

*SD* standard deviation.

In our study, esophageal adenocarcinoma was diagnosed in 6 individuals who underwent biopsies following the discovery of an esophageal polypoid lesion. Two of the individuals who were found to have duodenal polypoid lesions had a biopsy, which revealed duodenal adenocarcinoma. All malignant esophageal and duodenal lesions were ulcero-vegetative and fragile in appearance, and they were all assessed separately from benign esophageal lesions.

## Discussion

As the frequency of performing upper GI endoscopy increases in the world, the detection of non-gastric polyps has also increased. Although gastric polyps can be assessed more easily due to their frequency, non-gastric polyps cannot be recognized adequately due to their rarity. These polyps may be benign, as well as they may carry the risk of malignancy, and may be an indicator of an accompanying malignancy. In this study we sought to assess the prevalence of non-gastric polyps in the general population, their distribution by localization, their clinical significance, and follow-up and treatment approaches. Our research revealed that 194.52 out of 100,000 upper GI endoscopies discovered non-gastric polyps. When assessed according to polyp localization, the rate of esophageal polyp identification was 80.00 per 100,000, whereas the rate of duodenal polyp detection was 114.52 per 100,000. Squamous papilloma, inflammatory papilloma, and esophageal parakeratosis are the most frequently detected esophageal polyps (50.98, 16.47, and 4.70 per 100,000, respectively), whereas Brunner gland hyperplasia, inflammatory polyp, ectopic gastric mucosa, and adenomatous polyp are the most frequently detected duodenal polyps (32.16, 31.37, 16.47, and 15.69 per 100,000, respectively,).

Bulur et al.^22^ evaluated 19,560 patients and found non-gastric polyps in 38 of them. In our study, 127,493 patients were evaluated, and 248 non-gastric polyps were detected. Total evaluation revealed a detection rate of 194.52 non-gastric polyps per 100,000 patients. We were able to report the incidence rate in 100,000 patients as an epidemiological data for rare non-gastric polyps as a result of our study because of the large patient group we screened.

In the series of Szanto et al.^7^ evaluating 35-year upper gastrointestinal endoscopies, nearly 60,000 upper GI endoscopy was performed, and squamous papilloma was detected in 155 patients. None of these have turned into malignancies. Mosca et al.^9^ examined 7618 upper GI endoscopy procedures and detected squamous papilloma in 9 patients. In our study, squamous papilloma was detected in 65 of 127,493 patients evaluated over a 12-year period. Looking at the studies in the literature, the rate of detecting squamous papilloma in upper GIS endoscopy has been reported between 0.045 and 0.26%^6,7,9^. Consistently with the literature, this rate was found 50.98 per 100,000 patients in our study. None of the patients developed malignancy during their mean follow-up of 3.2 years. In a case presented by Kostiainen et al.^23^, the patient had the symptoms of dysphagia and vomiting due to large squamous papilloma. In our study, 63 of 65 patients with squamous papillomas were asymptomatic, while squamous papilloma larger then 20 mm was detected in two patient who had intermittent nausea and vomiting.

Mandard et al.^24^ found accompanying esophageal parakeratosis in approximately 40% of 400 patients, newly diagnosed with head and neck squamous cell carcinoma. However, no malignancy was found to originate from the parakeratotic area in the esophagus. In our study, 4 of 6 patients (66.6%) with esophageal parakeratosis had a history of squamous cell head and neck cancer (larynx and hypopharynx). In the light of these findings, it would be appropriate to screen the patients for possible head and neck malignancies in the case of esophageal parakeratosis detected incidentally in upper GI endoscopy.

Esophageal adenomas typically present as wide islets of dysplasia rather than a single polyp and typically develop in the presence of Barrett's esophagus. In a case series presented by Wong et al.^13^, polypoid lesions developing on the ground of Barrett's esophagus, with dysplastic adenomas and adenocarcinomas found in the pathology were evaluated. In our study, esophageal adenoma was detected in four patients and all four also had Barrett's esophagus. These patients underwent surgical esophagectomy afterward. The risk of adenocarcinoma is quite high in patients with Barrett's esophagus and accompanying adenoma in the esophagus, and these patients should be evaluated further, and the lesion should be removed endoscopically or surgically. Due to the presence of Barrett's esophagus in these patients, mucosectomy or ablation procedures for the disease should be integrated into endoscopic treatment strategies in addition to polyp excision. An esophagectomy is a surgical option in which the patient's polyp and esophagus segment are removed together, and the small intestine or stomach is anastomosed.

Levine et al.^25^ evaluated 27 patients with Brunner gland hyperplasia detected in the duodenum, and GIS bleeding was found in 10 of the patients, obstruction in 10, and incidentally in 7 patients. In our study, Brunner's gland hyperplasia was detected in the duodenum in 41 patients with 21 of them having GI bleeding and 9 signs of obstruction. It was detected incidentally in 11 of them. Although Brunner's gland hyperplasia are benign lesions, they should be treated as they may have clinical symptoms. We treated all patients endoscopically, except for one patient with obstruction. In the long-term follow-up, the patients were observed up as stable.

Ectopic gastric mucosa are benign lesions that can be detected incidentally in the duodenum. Naguchi et al.^19^ found ectopic gastric mucosa in 76 (55%) of 137 patients with duodenal ulcer, and *Helicobacter pylori* was detected in 59% (45/76) of the biopsies taken from them. In our study, duodenal ulcer was detected in 12 (57.1%) of 21 patients with ectopic gastric mucosa, and HP positivity was observed in 13 (61.9%) of them.

Sporadic duodenal adenomas are very rare and have the potential to transform into adenocarcinoma by showing similar morphological and molecular features with colorectal adenomas. The majority of sporadic duodenal adenomas are flat or sessile solitary lesions with pearly villi surfaces that develop on the descending duodenum's posterior or lateral walls^26^. Witterman et al.^17^ showed that 42% of patients with duodenal villous adenoma had malignant cells. In addition, it was shown that the rate of detecting concomitant colon adenoma is increased in these patients. In our study, all 20 adenomas detected in the duodenum originated from the second part of the duodenum, and malignant cells were detected in 1 of 4 villous adenomas. Again, 17 (85.0%) of these patients had adenomatous polyps in the colon. When polyps are found in the duodenum, they should be removed via snare polypectomy, endoscopic mucosal resection (EMR), endoscopic submucosal dissection (ESD), or argon plasma coagulation (APC) ablation due to their potential for malignancy. Then again, based on these findings, it would be a reasonable approach to be careful in terms of malignancy in patients with adenomatous polyps in the duodenum and to perform colon screening in these patients.

With a bed capacity of 3600 patients and more than 300 endoscopic procedures carried out each day, our center has the title of largest institution in Turkey and one of the three largest institutes in all of Europe. In addition to being an epidemiological study with a large patient group to evaluate, our study also offers suggestions for the best examination and treatment approaches to use when non-gastric polyps are found. The epidemiological study we conducted is the largest on non-gastric polyps ever published in the world's literature.

In conclusion, nowadays, with the widespread utilization upper GI system endoscopy, it is critical to recognize, monitor, and treat common lesions as well as less frequent but clinically significant lesions. Our study is not only the broadest evaluation of non-gastric polyps, but it also provides clinical approach recommendations for these polyps and discloses the prevalence of these polyps in the general population.

## Data Availability

The datasets used and/or analyzed during the current study available from the corresponding author on reasonable request.
